# Urban–rural disparities in minimum acceptable diet intake among children aged 6–23 months in Ethiopia: A multivariable Decomposition analysis of Ethiopian demographic and health survey 2019

**DOI:** 10.3389/fpubh.2024.1361673

**Published:** 2024-07-17

**Authors:** Anissa Mohammed, Abiyu Abadi Tareke, Awoke Keleb, Natnael Kebede, Yawkal Tsega, Abel Endawkie, Shimels Derso Kebede, Kaleab Mesfin Abera, Eyob Tilahun Abeje, Ermias Bekele Enyew, Chala Daba, Lakew Asmare, Fekade Demeke Bayou, Hussien Endris, Mastewal Arefaynie

**Affiliations:** ^1^Department of Epidemiology and Biostatistics, School of Public Health, College of Medicine and Health Science, Wollo University, Dessie, Ethiopia; ^2^Amref Health in Africa, COVID-219 Vaccine/Expanded Program for Immunization (EPI) Technical Assistant at West Gondar Zonal Health Department, Gondar, Ethiopia; ^3^Department of Environmental Health, College of Medicine and Health Sciences, Wollo University, Dessie, Ethiopia; ^4^Department of Health Promotion, School of Public Health, College of Medicine and Health Sciences, Wollo University, Dessie, Ethiopia; ^5^Department of Health System and Management, School of Public Health, College of Medicine and Health Sciences, Wollo University, Dessie, Ethiopia; ^6^Department of Health Informatics, School of Public Health, College of Medicine and Health Sciences, Wollo University, Dessie, Ethiopia; ^7^Department of Anaesthesia and Critical Care, College of Medicine and Health Science, University of Gondar, Gondar, Ethiopia; ^8^Department of Reproductive and Family Health, School of Public Health, College of Medicine and Health Sciences, Wollo University, Dessie, Ethiopia

**Keywords:** minimum acceptable diet, decomposition, EDHS, Ethiopia, urban–rural

## Abstract

**Introduction:**

The achievement of the minimum acceptable diet intake (MAD) stands at 14% among urban and 10% among rural under-five children in Ethiopia. Consequently, identifying the determinants of the urban–rural gap is vital for advancing Sustainable Development Goals (SDGs), fostering healthier communities, and developing evidence-driven approaches to enhance health outcomes and address disparities.

**Objective:**

The objective of the study was to decompose the urban–rural disparities in minimum acceptable diet intake in Ethiopia using the Ethiopian Mini-Demographic and Health Survey 2019 data.

**Method:**

The study was conducted using the Ethiopian Demographic and Health Survey 2019 report. A total of 1,496 weighted children aged 6–23 months were included using stratified sampling techniques. The main outcome variable minimum acceptable diet was calculated as a combined proportion of minimum dietary diversity and minimum meal frequency. A decomposition analysis was used to analyze the factors associated with the urban–rural discrepancy of minimum acceptable diet intake, and the results were presented using tables and figures.

**Result:**

The magnitude of minimum acceptable diet among children aged 6–23 months in Ethiopia was 11.0%. There has been a significant disparity in the intake of minimum acceptable diet between urban and rural under-five children with 14 and 10%, respectively. Endowment factors were responsible for 70.2% of the discrepancy, followed by 45.1% with behavioral coefficients. Educational status of college and above was responsible for narrowing the gap between urban and rural residents by 23.9% (β = 0.1313, 95% CI: 0.0332–0.245). The number of children in the household and the age of the child between 18 and 23 months were responsible for widening the gap in minimum acceptable diet intake discrepancy between urban and rural residents by 30.7% and 3.36%, respectively (β = −0.002, 95% CI: −0.003 to −0.0011 and β = −30.7, 95% CI: −0.025 – −0.0085). From the effect coefficients, the effect of institutional delivery was responsible for 1.99% of the widening of the gap between urban and rural residents in minimum acceptable diet intake (β = −0.0862, 95% CI: −0.1711 – −0.0012).

**Conclusion:**

There is a significant variation between urban and rural residents in minimum acceptable diet. The larger portion of the discrepancy was explained by the endowment effect. Educational status of mothers with college and above, parity, age of child, and place of delivery were the significant factors contributing to the discrepancy of minimum acceptable diet intake between urban and rural residents.

## Introduction

Widespread child undernutrition is a significant problem in low- and middle-income countries (1). According to the World Health Organization (WHO), the primary forms of undernutrition include wasting, stunting, and being underweight. In 2022, globally, 149 million children under 5 years old were stunted, and 45 million under 5 years old were wasted. Nearly half of the deaths among children under 5 years of age are linked to undernutrition, mostly occurring in low- and middle-income countries (2). In 2020, the global prevalence of stunting and underweight was 149 and 45 million, respectively (3). In developing countries, undernutrition contributes to 45% of mortality in children under 5 years old (4). In Ethiopia, the prevalence rates for wasting, underweight, and stunted growth among children under 5 years old were 7, 21, and 37%, respectively (5).

Malnutrition in children poses a substantial public health concern, particularly in low- and middle-income countries (6). Across the world, malnutrition is a direct or indirect factor in 60% of deaths among children under 5 years old, and inappropriate feeding practices are responsible for two-thirds of these fatalities (7). Inadequate infant and young child feeding (IYCF) practices are major determinants of undernutrition, affecting optimal growth and development, especially in the first 2 years of life (8). Exclusive breastfeeding provides essential nutrients and energy during the first 6 months. However, as children grow, exclusive breastfeeding alone is insufficient for their energy and nutrient needs (9). The World Health Organization (WHO) recommends introducing appropriate complementary foods alongside breastfeeding after 6 months. This combination of solid, semisolid, or soft foods and breastfeeding supports healthy child development after 6 months (8).

Proper complementary feeding, as recommended by the WHO, encompasses the introduction of solid, semisolid, or soft foods; compliance with minimum meal frequency (MMF) and dietary diversity; attainment of a minimum acceptable diet; and inclusion of iron-rich or iron-fortified foods (8). Minimum acceptable diet (MAD) encompasses both the percentage of children aged 6–23 months who obtain adequate dietary diversity and the required minimum meal frequency. Minimum dietary diversity serves as an indicator of sufficient micronutrient density in foods, defined as the proportion of children aged 6–23 months who received at least five out of eight specified food groups. Likewise, MMF denotes the proportion of children aged 6–23 months who received solid, semisolid, or soft foods a minimum number of times, acting as a proxy for fulfilling energy requirements (5). A meta-analysis conducted in Ethiopia revealed that the prevalence of MAD among children aged 6–23 months in urban areas was 47%, while it was 15% in rural areas (10). In addition, the Ethiopian Demographic and Health Survey 2019 reported a 14 and 10% intake of MAD in urban and rural areas, respectively (5). Furthermore, a study conducted in Pakistan revealed a significant difference in MAD intake between urban and rural children aged 6–23 months with 14 and 12%, respectively (11). Another study conducted in an urban slum area in India revealed a 2.4% achievement in MAD intake (12).

Both unfavorable child feeding practices and heightened infection rates pose risks to the health and development of children below 2 years of age (13). The repercussions of nutritional deficiencies during this early phase may manifest as childhood undernutrition of stunting and wasting (3). The major contributing factor to the problems was linked to inappropriate complementary feeding practices (5). In a bid to eliminate child undernutrition by 2030, the Ethiopian government has endeavored to enhance child feeding practices. This effort is manifested through the execution of the National Nutrition Program for Child Feeding Practice and a comprehensive multi-sectoral nutrition intervention plan (14). Furthermore, a pivotal strategy in this pursuit is the recently sanctioned 2019 Food and Nutrition Policy. The policy aims to attain optimal nutritional wellbeing across the life cycle by orchestrating the concentrated implementation of both nutrition-specific and nutrition-sensitive interventions. The focal point of these endeavors revolves around the effective implementation of various activities aimed at fostering improved nutritional outcomes (15).

Many factors have been identified in different studies that are associated with MAD. Child's age (11, 16–18), child sex (17), maternal educational status (17, 19), wealth index (20), antenatal care (21), women involvement in decision-making (22), number of children in the household (16, 23), institution delivery (20, 22, 24), and postnatal care (17, 25) were some of the identified factors associated with MAD intake. While numerous studies in Ethiopia have investigated the factors influencing MAD intake, none have specifically addressed the urban–rural gap in MAD intake among covariates. Additionally, a systematic review and meta-analysis conducted in Ethiopia (10) focused solely on determining the prevalence of MAD intake without identifying the factors associated with it. Hence, the findings of the urban–rural discrepancy will be instrumental in achieving the Sustainable Development Goals (SDGs) in order to provide a universal framework for tackling global issues and guaranteeing a better and more sustainable future for all by 2030. To be more precise, SDG 2 is to put an end to hunger, attain food security and better nutrition, and support sustainable agriculture. SDG 3 is to guarantee healthy lifestyles and promote wellbeing for all people, regardless of age. It is also helpful to foster healthier communities, to allow policy-makers to implement targeted interventions, and to enable the development of evidence-based strategies to improve health outcomes and reduce inequalities. Hence, this study aimed at decomposing the urban–rural disparities in MAD intake using the Ethiopian Mini-Demographic and Health Survey 2019 data.

## Methods

### Study design, setting, and period

The Ethiopian Mini-Demographic and Health Survey 2019 data set obtained from https://www.dhsprogram.com/data/dataset_admin/login_main.cfm website was used. A community-based cross-sectional study was conducted across urban and rural areas of Ethiopia between 21 March 2019 and 28 June 2019, as part of the Ethiopian Mini-Demographic and Health Survey (EMDHS) 2019, marking the second implementation of EMDHS in Ethiopia. The initial survey took place in 2014. Ethiopia, situated in the Horn of Africa, is located between latitudes 30 and 150 and 480 east. Encompassing a total area of 1,100,000 square kilometers, it comprises 11 ethnically and politically autonomous regional states, in addition to two administrative cities. The country has also witnessed rapid population growth, soaring from 53.5 million in the 1994 census to 114,968,588 in 2020, accompanied by a fertility rate of 4.3.

### Population and eligibility criteria

The source population for this study was all children aged 6–23 months in Ethiopia, while the study population was all children aged 6–23 months in the selected enumeration areas. Mothers with multiple children were surveyed about their youngest child within the 2 years preceding the study.

### Sample size and sampling techniques

A total of 1,496 weighted children aged 6–23 months were included in this study. Sampling weight was applied to ensure representativeness, addressing the non-proportional allocation of sample sizes across different regions and the urban and rural variations within them. Additionally, potential differences in response rates were considered. A total of 21 sampling strata were established. Enumeration areas (EAs) served as the sampling units for the initial stage of sampling. To ensure uniform survey precision across regions, sample allocation was carried out using an equal allocation method, resulting in the selection of 25 EAs from eight regions. However, 35 EAs were selected for each of the larger regions such as Amhara, Oromia, and the Southern Nations, Nationalities, and Peoples' Region (SNNPR). Overall, 305 EAs (93 in urban areas and 212 in rural areas) were chosen in the first stage, with selection probabilities proportional to EA size based on the 2019 EPHC frame (5, 26). In the EMDHS 2019, each region underwent stratification into urban and rural areas, forming 21 sampling strata. The sample was selected in two stages: first, stratified samples of census enumeration areas (EAs) in urban and rural zones were chosen, employing systematic probability sampling based on the sampling frame derived from all census EAs established for the 2019 Ethiopian population and housing census (EPHC), conducted by the Central Statistics Agency (CSA), and second, households (HH) were selected using the same systematic probability sampling method within the chosen EAs. Within each selected household, reproductive-age women with children under 2 years old were interviewed using an individual questionnaire.

### Data collection tools and variables

The World Health Organization (WHO) 2008 indicators for assessing infant and young children feeding practices were used to measure MAD. The MAD is a combined measure of minimum dietary diversity (MMD) and MMF.

1. The MDD signifies the percentage of children aged 6–23 months who were provided with five out of eight food groups (breast milk, grains, roots, and tubers, legumes and nuts, dairy products, flesh foods, eggs, and vitamin A-rich fruits and vegetables).
2. MMF indicates the proportion of breastfed children who were given solid, semisolid, or soft foods at least twice a day for infants aged 6–8 months and at least three times a day for children aged 9–23 months. For non-breast feed children aged 6–23 months, it involves receiving solid or semisolid food or milk feeds at least four times a day, with at least one of the meals being solid, semisolid, or soft food (26).
3. MAD indicates the proportion of children aged 6–23 months who fulfilled MDD and MMF.

Due to varying MMF cutoff values across age groups, MMF was computed for each age group separately, followed by the calculation of the overall meal frequency.

Once MMF and MDD were computed, children who satisfied both MMF and MDD criteria were classified as having met the MAD requirements. Conversely, if only one or neither criterion was met, they were categorized as not fulfilling the MAD criteria. Area of residence was the main predictor variable, categorized as urban and rural. Age of the mother and child, educational status of the mother, sex of the child, marital status, number of under-five children in the household, total number of children, twin sibling, region, wealth index, ANC, place of delivery, postnatal check, exclusive breastfeeding, duration of breastfeeding, current pregnancy, and immunization were the explanatory factors. The data were collected through a face-to-face interview with the mother or caregiver.

### Data analysis

The data were analyzed using STATA v17.0. Weighted frequencies and percentages were computed to address the non-response rate and design effect of DHS data. Descriptive statistics, encompassing frequencies with percentages, mean, and standard deviation, were reported, and the results were presented using tables and graphs. The multivariable Oaxaca decomposition analysis was used to analyze the urban–rural disparity of MAD intake among children aged 6–23 months. This method utilizes the output of regression models, such as mean or proportion, to dissect into a component attributable to compositional differences between groups. The urban–rural discrepancy in MAD intake was decomposed into the endowment effect (contribution of respondents' characteristics and their environment) and the coefficient effect (the response to behavior) or the interaction of the two. The difference in MAD was ascribed to a gap in endowments (E), coefficients (C), or the interaction of endowments and coefficients. The Oaxaca decomposition uses the high group (urban children) as the reference group, with weighting contrasts in attributes by the coefficients of urban children and contrasts in coefficients by the covariates of rural children (27, 28).

If Yi, was the outcome variable and an independent variable X, and we have two groups, urban and rural, then the MAD for the rural and urban women is given as ε.


$$\begin{array}{c} Y_{\text{i}}^{\text{rural}}=\beta^{\text{rural}}X_{\text{i}}+\;\varepsilon^{\text{rural}}\\ Y_{\text{i}}^{\text{urban}}=\beta^{\text{urban}}X_{\text{i}}+\;\varepsilon^{\text{urban}} \end{array}$$

Thus, the urban–rural gap in the mean MAD intake (Y^urban^ – Y^rural^) is given as follows:


$$\begin{array}{l} Y^{\text{urban}}-Y^{\text{rural}}=\left(X^{\text{urban}}-X^{\text{rural}}\right)\;\beta^{\text{rural}}+\left(\beta^{\text{urban}}-\beta^{\text{rural}}\right)\;X^{\text{urban}}\\ \;+\;\left(X^{\text{rural}}-X^{\text{urban}}\right)\left(\beta^{\text{rural}}-\beta^{\text{urban}}\right)\\ Y^{\text{urban}}-Y^{\text{rural}}=\Delta X\beta^{\text{urban}}+\Delta \beta X^{\text{rural}}+\Delta X\Delta \beta \\ =E+C+CE \end{array}$$

where ΔX is the mean difference of the explanatory variables (X^urban^ – X^rural^).

A coefficient with a 95% confidence interval and a *p*-value of 0.05 was utilized to establish statistical significance.

## Results

### Socio-demographic characteristics

A total of 1,496 weighted participants were included in this study. The mean age of the mothers was 27.6 (SD = 6.3), with majority being in the age group of 25–34 years in urban (226, 52.8%) and rural (525, 49.2%) residents. A significant portion of participants (553, 51.8%) in urban areas were unable to read and write, while 425 (39.8%) attended primary education. Children aged 12–18 months were most prevalent in both urban (204, 47.7%) and rural (448, 41.9%) residents. Furthermore, majority of the households had three or less under-five children in both urban (421, 98.8%) and rural (1,054, 98.8%) residents. Regarding the wealth status, more than three-fourths of the participants in urban areas (346, 81%) were rich in wealth, while more than half of rural residents (555, 52%) were poor in wealth (Table 1).

**Table 1:** Maternal, socio-economic, and demographic characteristics of women participants based on place of residence using EDHS 2019 mini-report.

**Variables**	**Category**	**Urban**	**Rural**
Age of the mother	15–24	125 (29.1%)	350 (32.8%)
	25–34	226 (52.8%)	525 (49.2%)
	35–49	77 (18.1%)	193 (18.1%)
Educational status of mother	Unable to read and write	112 (26.2%)	553 (51.8%)
	Primary education	197 (46.1%)	425 (39.8%)
	Secondary education	58 (13.6%)	67 (8.4%)
	College and above	60 (14.1%)	23 (2.2%)
Age of child	6–11 months	116 (27.1%)	361 (33.8%)
	12–18 months	204 (47.7%)	448 (41.9%)
	19–24 months	108 (25.2%)	259 (24.2%)
Sex of child	Male	224 (52.4%)	548 (51.3%)
	Female	204 (47.7%)	520 (48.7%)
Sex of household head	Male	328 (76.5%)	962 (90.0%)
	Female	101 (23.5%)	107 (9.9%)
Marital status	Married	406 (94.9%)	1,037 (97.2%)
	Not married	22 (5.1%)	30 (2.8%)
Number of under-five children	≤ 3	421 (98.2%)	1,054 (98.7%)
	4–5	8 (1.8%)	13 (1.3%)
Total number of children	≤ 3 children	305 (71.2%)	592 (55.4%)
	4–6 children	81 (18.9%)	337 (31.6%)
	>6	42 (9.8%)	139 (13.0%)
Twin child	No	414 (96.7%)	1,054 (98.6%)
	Yes	14 (3.3%)	15 (1.4%)
Region	Agrarian	327 (76.4%)	974 (91.2%)
	Pastoralist	47 (11.0%)	90 (8.4%)
	Urban	54 (12.5%)	4 (0.4%)
Wealth index	Poor	59 (14%)	555 (52%)
	Middle	23 (5%)	259 (24%)
	Rich	346 (81%)	255 (24%)
Antenatal care	Yes	335 (81.0%)	631 (60.2%)
	No	78 (19.0%)	417 (39.8%)
Place of delivery	Home	101 (23.8%)	548 (51.3%)
	Institution	327 (76.3%)	520 (48.7%)
Post natal check within 2 months	No	343 (83.0%)	920 (87.8%)
	Yes	70 (17.0%)	128 (12.2%)
Exclusive breastfeeding	No	347 (85.4%)	890 (88.1%)
	Yes	59 (14.6%)	121 (11.9%)
Duration of breastfeeding	Ever breastfeeding	74 (17.2%)	134 (12.6%)
	Still breastfeeding	347 (81.2%)	898 (84.0%)
	Never breastfeed	7 (1.6%)	36 (3.4%)
Current pregnancy	No	409 (95.6%)	1,003 (94.9%)
	Yes	19 (4.4%)	65 (6.1%)

### Maternal-related characteristics

More than three-fourths of urban residents had antenatal care follow-up (335, 81.0%) and had an institution delivery (327, 76.3%), while more than half of rural residents (548, 513%) had home delivery. Less than one-quarter of urban (70, 17.0%) and rural residents (128, 12.2%) had postnatal check within 2 months. More than three-fourths of urban (347, 81.2%) and rural (898, 84.0%) residents were still breastfeeding (Table 1).

### Magnitude of minimum acceptable diet

**Figure 1:**
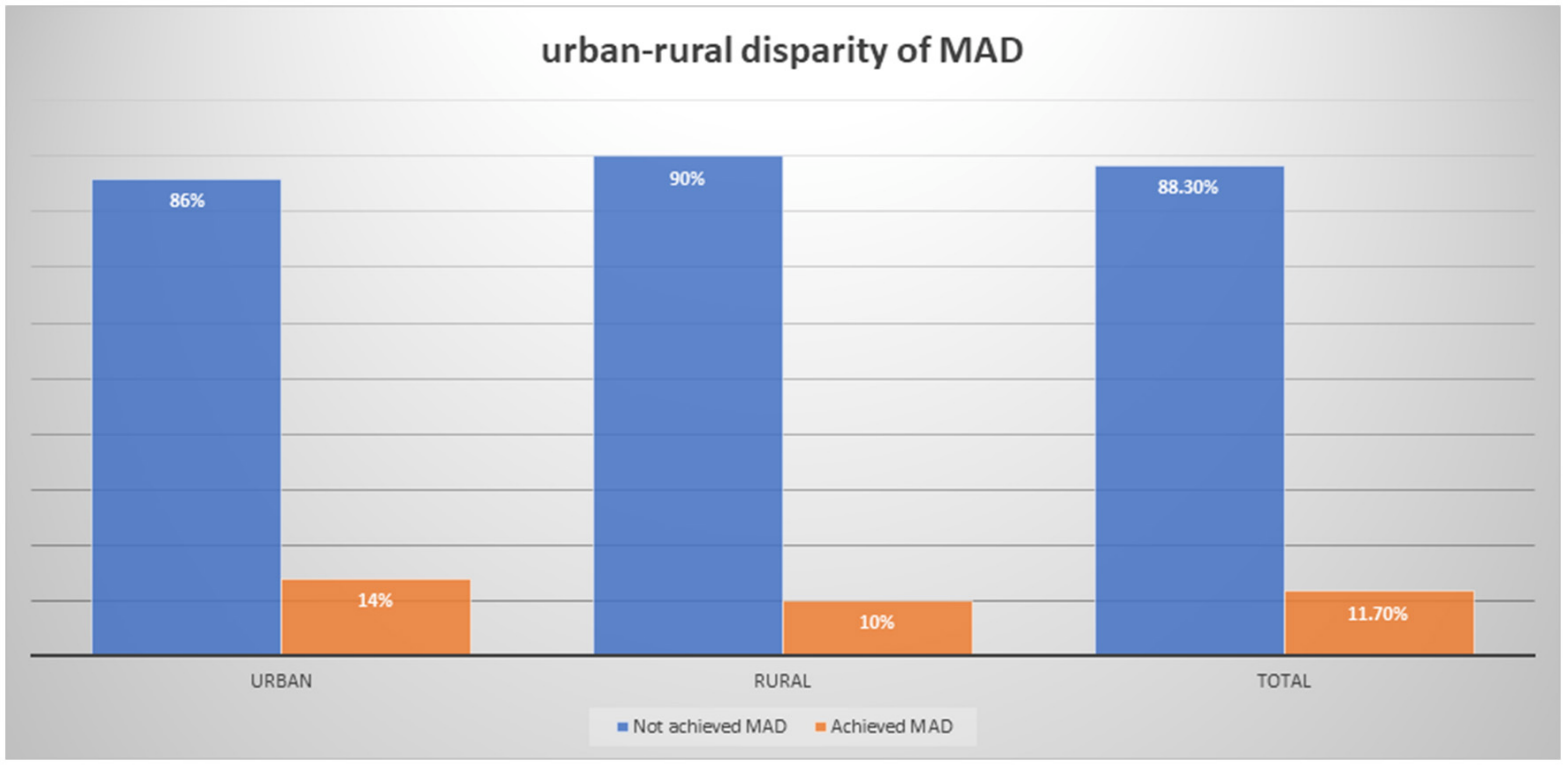
Urban–rural disparity of MAD intake among children aged 6–23 months using EDHS 2019.

**Figure 2:**
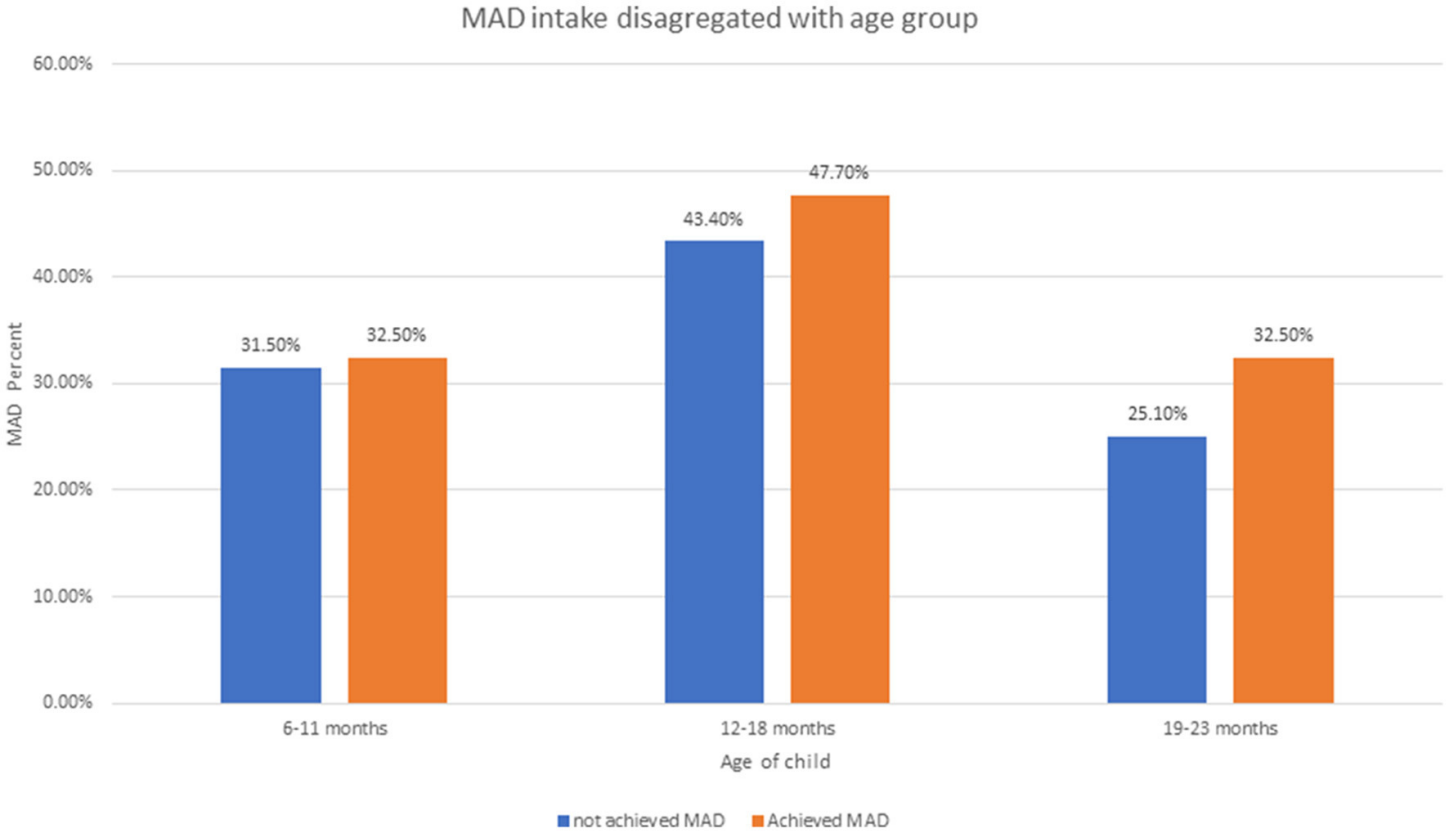
Minimum acceptable diet intake disaggregated by children aged 6–23 months using EDHS 2019.

The proportion of MAD among children aged 6–23 months was 11.7% (95% CI: 10.1–13.5). The magnitude of MAD in urban residents was higher than that in rural residents with 14 and 10%, respectively (Figure 1). Similarly, the MAD was disaggregated by age and sex of the child. Only 44 (32%) of 6–11 months, 64 (47.7%) of 12–18 months, and 26 (19.8%) of 6–11 months children achieved the MAD intake (Figure 2). The minimum meal frequency was fulfilled for 59% of urban and 54% of rural residents. In total, 16 and 12% of urban and rural residents fulfilled the minimum dietary diversity, respectively.

### Decomposition analysis

Tables 2, 3 show the decomposition analysis. There was a significant difference or disparity in the uptake of minimum acceptable diet (MAD) between urban and rural residents in which urban residents had a 0.37 times higher uptake of MAD than rural residents. This disparity was explained mostly by the difference in endowment characteristics with 70.2% and *p*-value < 0.046. This indicates that if the respondents' characteristics in urban and rural women were similar, the gap related to uptake of MAD would be reduced by 70.2%. The change in the effect of endowment characteristics among infants and young children whose mothers attained college and above educational status, child's age between 19 and 23 years, and the number of under-five children above three were significantly attributed to the rural-urban disparity in the uptake of MAD. The difference in educational attainment between rural and urban women was the primary factor attributed to 23.95% of the gap in MAD (β = 0.0117, 95% CI: 0.0115, 0.0356). If the same proportion of rural women had a college education or higher as urban women, the difference between urban and rural areas in meeting the MAD would decrease by 23.95%. Additionally, variations in the number of children under 5 years old and the total number of children in households between rural and urban areas contributed to the widening gap in the adoption of the MAD (β = −0.0012, 95% CI: −0.0072 – 0.0252 and β = −0.036, 95% CI: −0.025 – −0.0085). If more families with young children (number of under-five children and total number of children) moved from rural areas to urban areas, we could reduce the gap in uptake of MAD by 0.49 and 3.36%, respectively. Furthermore, if there are more children aged 19–23 months in rural areas than in urban areas, the gap between the two areas would get bigger by 30.7% (β = −0.002, 95% CI: −0.003 – −0.0011).

**Table 2:** Endowment factors of decomposition analysis of urban–rural disparity of MAD intake among children aged 6–23 months from EDHS 2019.

	**Coefficient with 95% CI**	**Percent**	***p*-value**
Raw difference	0.37	100	0.000
Explained	0.27	70.2	0.046
Unexplained	0.09	45.1	0.003
**Endowment (explained characteristics)**			
**Age of the mother**			
15–24	1		
25–34	−0.003 (0.018 – 0.006)	−1.96	0.645
35–49	−0.001 (−0.003 – 0.002)	−0.04	0.403
**Educational status of mother**			
Unable to read and write	1		
Primary education	−0.00189 (−0.00271 – 0.00345)	−3.88	0.452
Secondary education	−0.0129 (−0.0286 – 0.0026)	43.89	0.675
College and above	0.1313 (0.0332 – 0.245)	23.95	**0.000** ^*^
**Age of child**			
6–11 months	1		
12–18 months	0.00052 (−0.0003 – 0.0.0014)	1.77	0.236
19–23 months	−0.002 (−0.003 – −0.0011)	−30.7	**0.003** ^*^
**Sex of child**			
Male	1		
Female	−0.0026 (−0.0025 – 0.0085)	−0.88	0.385
**Marital status**			
Married	1		
Not married	0.002 (−0.007 – 0.001)	22.5	0.776
**Number of under–five children**			
≤ 3	1		
>3	−0.0012 (−0.0072 – 0.0252)	−0.49	**0.009** ^*^
**Total number of children**			
≤ 3 children	1		
4 children	−0.036 (−0.025 – −0.0085)	−3.36	**0.023** ^*^
>6	−0.00181 (−0.0052 – 0.00165)	−6.12	0.305
**Place of delivery**			
Home	1		
Institution	0.01399 (−0.02761 – 0.00253)	43.89	0.534
**Postnatal check within 2 months**			
Yes	1		
No	−0.001 (−0.0022 – 0.0005)	−0.59	0.212

^*^Statistically significant at p < 0.05.

**Table 3:** Coefficient (behavioral) factors of the decomposition analysis of urban–rural disparity of MAD intake among children aged 6–23 months from EDHS 2019.

	**Due to difference in coefficient (E)**		
	**Coefficient (95% CI)**	**Percent**	***p*** **-value**
**Age of the mother**			
15–24	1		
25–34	−0.0283 (−0.065 – 0.008)	−95.5	0.129
35–49	−0.0090 (−0.025 – 0.007)	30.6	0.263
**Educational status of mother**			
Unable to read and write	1		
Primary education	−0.033 (−0.202 – 0.137)	−22.9	0.75
Secondary education	−0.0154 (−0.0279 – 0.0081)	−34.6	0.89
College and above	0.002 (0.001 – 0.008)	1.59	0.03^*^
**Age of child**			
6–11 months	1		
12–18 months	−0.0104 (−0.0269 – 0.0060)	35.8	0.214
19–23 months	−0.0053 (−0.02016 – 0.0095)	17.9	0.483
**Sex of child**			
Male	1		
Female	−0.05 (−0.02 – 0.081)	−34.7	0.071
**Marital status**			
Married			
Not married	−0.0103 (−0.0248 – 0.0042)	34.7	0.165
**Number of under-five children**			
≤ 3			
>3	0.02381 (−0.1930 – 0.2407)	80.5	0.83
**Total number of children**			
≤ 3 children			
4–6 children	−0.02171(−0.0682 – 0.0247)	−0.92	0.360
>6	0.0006 (−0.0115 - 0.0127)	0.10	0.923
**Place of delivery**			
Home	1		
Institution	−0.0862 (−0.1711 – −0.012)	−1.99	0.047^*^
**Postnatal check within 2 months**			
Yes	1		
No	−0.003 (−0.008 – 0.0024)	−1.08	0.280

The symbol * indicates significant level at *p* < 0.05.

Overall, 45% of the alteration between urban and rural MAD intake was accounted for due to differences in effect or coefficients. “Alongside the positive impact of higher education levels in reducing the urban–rural disparity in providing minimum acceptable diet (MAD) to children, the shift from home to institutional delivery among rural women widened the gap by 1.99% (β = −0.0862, 95% CI: −0.1711 - −0.0012). This means that if more rural women opted for institutional deliveries instead of giving birth at home, the disparity in MAD uptake between urban and rural areas would increase.”

## Discussion

The aim of this study was to assess the urban–rural disparities in the uptake of MAD among children aged 6–23 months in Ethiopia. The result revealed that while the uptake of MAD in urban areas was higher than that in rural areas, the decomposition analysis unveiled critical insights into the underlying factors driving this discrepancy. Most of the discrepancy is mainly due to endowment factors, meaning that when the coefficient effect is held constant, almost three-fourths of the disparity in uptake of MAD were due to differences in the distribution of MAD covariates; hence, the effect of composition factors (endowment) was found to be more significant in reducing the urban–rural gap. This can be explained by the fact that urban children had better uptake of MAD than rural residents because urban children were better endowed with the factors that trigger MAD take.

Attaining the highest level of education emerges as a key factor in reducing the gap in MAD uptake. This finding aligns with research conducted in various regions, including Uganda, Bangladesh, Sub-Saharan Africa, and Nepal (19, 23, 29, 30), where urban parents with college or higher education were more inclined to adopt MAD compared to their counterparts. This tendency can be attributed to their enhanced access to information via diverse channels, such as the Internet, leading to a better understanding of the significance of a balanced diet for children's wellbeing. Additionally, urban women typically enjoy greater economic prospects, higher living standards, and improved infrastructure, facilitating access to a diverse range of foods throughout the year. It has been supported in different studies that having better wealth status results in better uptake of MAD (19, 20, 31). This explanation was also supported by our study in the descriptive statistics, which found that 81% of urban residents compared with 24% of rural residents were rich in wealth. Not only the covariate effect of highest education influences MAD uptake gap but also the behavioral effect of highest education attainment plays a significant role in narrowing the gap of MAD uptake between urban and rural residents. This is supported by a study conducted in Bangladesh (30). Hence, the study offers actionable insights for policy-makers and public health practitioners seeking to address disparities in child nutrition and improve overall health outcomes in rural communities.

The disparity in MAD uptake between urban and rural areas is significantly influenced by the number of children under 5 years old and the total number of children in households. Our analysis suggests that if urban areas had a similar number of children aged 6–23 months as rural areas, the gap would decrease by 0.49 and 3.336%, respectively. Notably, rural households tend to have a higher percentage of families with more than four children compared to urban households (45 vs. 29%). Consequently, children with fewer than three siblings are more likely to receive MAD. It is in line with studies conducted in Nepal and Brazil (16, 23). If the number of under-five children and the total number of children in the household are large, there will be competition for financial resources, time, and food supplies, which makes it challenging for caregivers to fulfill the nutritional requirements of each child. Furthermore, taking care of multiple children demands a lot of energy, fatigue, and stress, which, in turn, affects parents' ability to prepare nutritious foods and ensures the child's uptake of MAD.

Additionally, children aged 19–23 months encounter difficulties transitioning their diet from child feeding practices to family foods, mainly solid foods, resulting in challenges in introducing new solid foods, and child may be resistant to testing a variety of food textures. Furthermore, parents may assume that the child is grown enough to consume family foods and may not be fully informed in preparing a variety of foods that fulfill the MAD of this aged children. This is similar to studies conducted in Addis Ababa, Pakistan, and Indonesia (11, 17, 18).

The alteration in the uptake of MAD between urban and rural residents is also explained by behavioral or effect factors, which is 45.1%. The coefficients of the unexplained factor imply that a change in covariates increasing MAD intake might result in marginal enhancement in MAD intake in rural areas, which is comparatively insignificant compared to urban areas. This indicates that increasing behavioral changes are crucial. It has been found that place of delivery was a significant factor in the discrepancy between urban and rural MAD intake; this is similar to studies conducted in Ethiopia in Goncha district (20), Dembecha district (22), and Gondar (32). Delivering in the health institution resulted in better access to healthcare information, postnatal care, and immunization. Furthermore, women of institutional deliveries often provide a healthcare professional who can guide and support mothers in various aspects of child care and nutrition support. For these reasons, redistribution of education, balancing of the number of children or controlling parity in the household, improving place of delivery, and improving overall determinants in rural areas might not be adequate to achieve the urban–rural gap. Hence, interventions aimed at narrowing the gap between urban–rural residents would necessitate the implementation of behavioral and awareness programs to enhance the impact of the existing determinants.

The current study using decomposition analysis offers valuable insights into the complex interplay of factors contributing to urban–rural disparities in MAD uptake, informing targeted interventions for reducing this gap. Furthermore, the results will be representative of the source population since the data are nationwide. However, the cross-sectional nature of the study limits its ability to establish a temporal relationship between variables.

## Conclusion

There is a significant variation between urban and rural residents in minimum acceptable diet intake. The larger portion of the discrepancy was explained by the endowment effect. Educational status of mothers with college and above, parity, age of child, and place of delivery were the significant factors in the discrepancy between urban and rural MAD intake. Therefore, it is recommended to improve educational programs for rural residents, targeting increasing awareness of children's minimum acceptable diet intake based on the recommended ages and focusing on providing information through different channels to narrow the gap between urban and rural residents. Besides, it is better to strengthen family planning and child spacing initiatives to ensure proper feeding practices by alleviating the financial competition in the household. Moreover, since the first Sustainable Development Goal (SDG) is to eradicate poverty, it is crucial for governments to implement comprehensive policies aimed at reducing these nutritional disparities. Such policies should focus on improving educational opportunities, economic conditions, and healthcare infrastructure in rural areas. By addressing these determinants, governments can not only enhance the nutritional status of young children but also contribute to broader goals of poverty reduction and improved public health. Implementing these policies will be instrumental in fostering healthier communities and achieving sustainable development in Ethiopia.

## Data availability statement

The raw data supporting the conclusions of this article will be made available by the authors, without undue reservation.

## Ethics statement

Since the study was a secondary data, the ethical procedures were conducted by the institution conducting the survey. However, we have acquired written permission from the measure DHS program, which granted authorization for the use of this data set. Before the data collection technique, the materials were reviewed and approved for compliance called “protection of human subjects” by the Institution Review Board (IRB). Strict confidentiality measures were adhered to ensuring anonymity, and the data was exclusively utilized for the specific purposes outlined in the current study. The studies were conducted in accordance with the local legislation and institutional requirements. Written informed consent for participation in this study was provided by the participants' legal guardians/next of kin.

## Author contributions

AM: Conceptualization, Data curation, Funding acquisition, Supervision, Validation, Writing – original draft, Writing – review & editing. AT: Conceptualization, Validation, Visualization, Writing – original draft, Writing – review & editing. AK: Conceptualization, Supervision, Validation, Visualization, Writing – original draft, Writing – review & editing. NK: Conceptualization, Supervision, Validation, Visualization, Writing – original draft, Writing – review & editing. YT: Conceptualization, Supervision, Validation, Visualization, Writing – original draft, Writing – review & editing. AE: Conceptualization, Supervision, Validation, Visualization, Writing – original draft, Writing – review & editing. SK: Conceptualization, Supervision, Validation, Visualization, Writing – original draft, Writing – review & editing. KM: Formal analysis, Investigation, Methodology, Writing – original draft, Writing – review & editing. ET: Formal analysis, Methodology, Software, Supervision, Writing – original draft, Writing – review & editing. EB: Investigation, Methodology, Software, Validation, Writing – original draft, Writing – review & editing. CD: Investigation, Validation, Visualization, Writing – original draft, Writing – review & editing. LA: Formal analysis, Software, Validation, Visualization, Writing – original draft, Writing – review & editing. FB: Conceptualization, Supervision, Validation, Visualization, Writing – original draft, Writing – review & editing. HE: Software, Supervision, Validation, Visualization, Writing – original draft, Writing – review & editing. MA: Software, Supervision, Validation, Visualization, Writing – original draft, Writing – review & editing.
